# *Hura crepitans* Seeds for Control of *Eimeria* spp. in Lambs as an Alternative to Conventional Therapies

**DOI:** 10.3390/vetsci9090488

**Published:** 2022-09-08

**Authors:** Martha-Yanira Velazquez-González, Jose-Lenin Loya-Olguin, Yissel-Sacnicte Valdes-Garcia, Sergio Martinez-Gonzalez, Fidel Avila-Ramos, Francisco Escalera-Valente, Jose-Ramiro Gonzalez-Montaña

**Affiliations:** 1 Ciencias Biológico-Agropecuarias, Universidad Autónoma de Nayarit, Xalisco 63780, Mexico; 2 Unidad Académica de Medicina Veterinaria y Zootecnia, Universidad Autónoma de Nayarit, Compostela 63700, Mexico; 3 División Ciencias de la Vida, Campus Irapuato-Salamanca, Universidad de Guanajuato, Guanajuato 36500, Mexico; 4 Departamento de Medicina, Cirugía y Anatomía Veterinaria, Facultad de Veterinaria, Universidad de León, 24007 León, Spain

**Keywords:** *Hura crepitans*, coccidiosis, antiprotozoal, natural antiparasitic, sheep, in vivo study

## Abstract

**Simple Summary:**

The anticoccidial effect of *Hura crepitans* seed was evaluated for 3 weeks. For this, 21 lambs were used, which were housed individually in raised wire-mesh pens with individual water troughs and feeders. The lambs were randomly divided into three groups (*n* = 7) that received different doses of *Hura crepitans* seeds. The treatment was administered at the beginning of the experiment once orally. Faecal samples were taken for quantitative analysis of oocysts and blood for haematological and biochemical analyses. Lambs that received the seeds showed significant excretion of coccidia oocysts between days 5 and 7 post-treatment, without affecting feed intake and growth. Most blood parameters analysed showed values within the reference interval, while urea and BUN levels increased slightly but without statistical difference. We can conclude that *Hura crepitans* seeds, at doses of 4 and 6 g/kg, favour the expulsion of coccidia oocysts, without affecting either the health or the productive behaviour of lambs.

**Abstract:**

To evaluate the anticoccidial effect of the *Hura crepitans* seed, 21 cross-breed lambs, 23 ± 3 kg live weight (PV) and 70 ± 6 days old, were used. Animals were housed individually in raised wire-mesh pens, with individual water troughs and feeders. Lambs were randomly divided into three groups (*n* = 7) that received different doses of *Hura crepitans* seeds: 4 g/kg B.W. (group 1), 6 g/kg B.W. (group 2) and 0 g/kg B.W. (control group or group 0). The treatment was administered at the beginning of the experiment once orally and the study lasted 21 days. Stool samples were taken for quantitative analysis of oocysts using the McMaster technique and blood for haematological and biochemical analysis. Lambs that received the seeds showed a higher excretion of coccidia oocysts between days 5 and 7 after treatment (*p* < 0.05) due to the antiparasitic effect of these seeds, without affecting their productive performance (daily feed intake (DFI)- total weight gain (TWG), daily weight gain (DWG)). In addition, the daily feed consumption (DFC) and dry matter intake (DMI) were similar between the groups. The analysed enzymes (ALT, AST, GGT and FA) and creatinine showed reference values. Only the urea and BUN levels were slightly increased, but without statistical difference between the groups. Therefore, the *Hura crepitans* seed, at doses of 4 and 6 g/kg, favours the expulsion of coccidial oocysts, without affecting either the health or the productive behaviour of lambs.

## 1. Introduction

Parasitic diseases are an important factor in livestock productivity in Mexico [1]. In sheep, the main parasites that affect the gastrointestinal system are helminths and protozoa [2]. Coccidiosis is a disease caused by the presence of protozoa of the *Eimeria* genus [3,4], which affects sheep of all ages, although the youngest animals are the most susceptible to developing the disease [5]. 

The economic losses produced by coccidiosis in small ruminants are the result of the high mortality rate in lambs and kids and low weight gain, which, therefore, leads to low productivity [6].

There are not enough data on the economic impact of this disease, and there are even less for tropical regions and small ruminants. Therefore, the economic losses it causes cannot be estimated for either clinical or subclinical diseases [7]. Fitzgerald, however, in 1980, estimated an annual global loss of $140 million due to coccidia infections in sheep. In Mexico, the economic impact caused by *Eimeria* spp. in bovine cattle is 23.78 million USD, while in the USA, annual economic losses due to eimeriosis have been estimated at USD 62 million [1]. Thus, in the USA, it has been estimated that 77 million young cattle were infected with coccidia during the first year of life, of which 4 million would be treated against eimeriosis, with around 80,000 animals dying [1].

Among the different species that affect sheep, *Eimeria crandallis* and *Eimeria ovinoidalis* are considered very pathogenic, since they are associated with clinical cases of coccidiosis in lambs, parasitizing the distal part of the intestine (thin and thick) of young animals [8]. *Eimeria ovinoidalis* causes destruction of the intestinal villi, which leads to denudation of the cecal mucosa, resulting in haemorrhagic enteritis, dehydration, shock and/or death [8].

Traditionally, coccidiosis in lambs is treated with sulfonamides for two or three days or coccidiostats, such as amprolium, monensin, decoquinate, diclaruzil, toltrazuril, etc. [9,10]. However there is currently an increase in resistance to multiple products that are frequently used for the prevention and treatment of coccidiosis [11,12,13].

This is due to multiple causes, including frequency of treatment and underdosing [14]. Thus regarding helminths, deworming animals regularly, or using the same anthelmintic group, favours the appearance of parasitic resistance [15], while underdosing contributes to the survival of resistant worms, favouring the selection of resistant strains [15].

Due to the increasing reports concerning the appearance of parasitic resistance against commonly used drugs worldwide, the development of new control strategies is required [14,16]. Castagna et al. [17] points out that many plants have been used in ethnoveterinary medicine for parasite control in small ruminants. In this context, the administration of methanolic extracts from the *Ruta pinnata* plant has been evaluated in vitro in sheep, and it was found to show activity against *Eimeria ninakohlyakimovae* by inhibiting sporulation of oocysts and decreasing the viability of sporozoites. Furthermore, its use has even been postulated as a treatment against other parasites in animals and humans [18]. Saratsis et al. [19] found a reduction in the load of *Eimeria* oocysts in lamb, with the use of *Onobrychis viciifolia*, a plant known as sainfoin that is cultivated as a forage plant in various countries. *Sericea lespedeza* has also been shown to be effective in the prevention and control of coccidiosis, as well as in the reduction of gastrointestinal nematode infection, reducing the loss of lambs after weaning [20]. In southern Italy, in Calabria, *Punica granatum* macerate has been verified as having significant anthelmintic effects, while, in contrast, low effectiveness was found for the *Artemisia campestris* macerate and *Salix caprea* macerate was judged to be completely ineffective [17]. Moreover, *Artemisia absinthium*, or wormwood, although it has been traditionally used as a febrifuge and anthelmintic, does not seem to have a significant effect against coccidiosis [10].

*Hura crepitans* (Linneo, 1753), also called sandboxtree or possumwood, is an evergreen tree of the Euphorbiaceae family, native to tropical regions of America and widely distributed throughout the world. Although it is poisonous to humans, sheep routinely consume their seeds and, according to the popular belief of farmers, they do so to deworm. 

Phytochemical evaluation has shown that *Hura crepitans* contains multiple substances (tannins, saponins, flavonoids, coumarins, glycosides and triterpenoids). The presence of these phytochemicals confirms the many applications of *H. crepitans* in traditional medicine [21], where it has been used as a purgative, emetic, antimicrobial, anti-inflammatory and hepatoprotective agent. It has also been used in the treatment of mucous diarrhoea in dogs and humans [21,22,23]. The latex of the *Hura crepitans* seed (sHC) has been used to manage skin diseases in humans [24] and as an intestinal antiparasitic [25], and it has even been used successfully in sheep as an intestinal antiparasitic against *Haemonchus contortus* and *Strongyloides venezuelensis* [26]. 

However, we are not aware of any evaluation of the use of sHC in the control of coccidia in ruminants. Likewise, we do not know the effects of its use on the health of animals, since some toxic plants are potentially harmful to specific organs and systems, causing tissue damage [27]. Therefore, the aim of the present research was to evaluate the effects of different doses of *Hura crepitans* seeds for control of coccidiosis in lambs, as well as their effect on the performance and health of animals.

## 2. Materials and Methods

### 2.1. Study Location

The research was carried out in the Unidad Académica de Medicina Veterinaria y Zootecnia, Universidad Autónoma de Nayarit (UAMVZ-UAN), geographically located between 21°17′46″ north latitude and 104°54′ west longitude, at 880 m (a.s.l.). The area has a semi-warm and humid climate, with an average annual temperature of 22 °C and precipitation of 1000 mm [28]. 

The handling of lambs complied at all times with the approved regulations for the use and welfare of animals (NOM-051-ZOO-1995: Humanitarian care of animals during mobilization of animals [29]; NOM-062-ZOO-1999: Technical specifications for the care and use of laboratory animals). Livestock farms; farms; centres of production, reproduction and breeding; zoos; and exhibition halls must meet the basic requirements for animal welfare [25,30], and the experimental protocol was approved by the corresponding Committee of the Universidad Autónoma de Nayarit, Tepic, México.

### 2.2. Obtaining the Seeds

The whole, ripe fruits of *Hura crepitans*, characterized by a brown colour on their skin [26,31], were collected within the UAMVZ-UAN during the months of April and May. The shells of the fruit were broken with a hammer and, subsequently, the seeds were broken into four pieces and ground using an Oster^®^ blender; finally they were dehydrated at 65 °C in a forced air oven for 48 h.

### 2.3. Experimental Groups

Twenty-one female Pelibuey x Katahdin cross lambs, 70 ± 6 days olds, weighing 23 ± 3 kg B.W., with a mean body condition score (BCS) of 3/5, were used [32]. They had been weaned at approximately 2 months and had a period of adaptation to food and housing prior to the start of the experiment. The animals were housed individually in raised pens with a wire mesh, with individual water troughs and feeders.

Lambs were randomly assigned to the different experimental groups (Gr. 1, Gr. 2 or Gr. 0). Each group (*n* = 7) received a different amount of *Hura crepitans* seeds: 4 g/kg B.W. (in Gr. 1), 6 g/kg B.W. (in Gr. 2) and 0 g/kg B.W. (the Control Group, called Gr. 0). The treatment was administered orally once at the beginning of the experiment. The study lasted for 21 days.

### 2.4. Feed

All animals received the same ration, formulated according to the recommendations of the NRC [33], which was offered ad libitum (Table 1). Dry matter intake (DMI) was calculated based on daily feed intake (DFI). For this, both the food offered and the rejected food were weighed and recorded every day. Animals were weighed prior to the first morning feeding, at the beginning and at the end of the experiment, to calculate the total weight gain (TWG). Feed conversion efficiency (FCE) was calculated based on the TWG and DMI.

### 2.5. Sampling Blood and Stool

Faecal samples were taken from each lamb on days 0, 1, 2, 3, 4, 5, 7, 9, 11, 15 and 21 of the experiment and blood samples on days 0, 1, 3, 7, 9 and 11. The first sampling (day 0) was carried out before the administration of seeds. The faeces (approx. 20 g) were collected directly from the rectum, placed in sterile plastic bags (Whirl-Pak Nasco) and kept refrigerated until processing, which always took place within one week. 

The quantitative analysis of coccidial oocysts per gram of faeces (opg) was carried out with the McMaster technique [34] in the Laboratorio de Parasitología de la UAMVZ-UAN. A subsample consisting of the samples taken on day 0 from all the animals was sent to the Laboratorio de Parasitología of the Facultad de Medicina Veterinaria y Zootecnia de la Universidad Nacional Autónoma de México (FMVZ-UNAM) to identify the Eimeria species, taking into account the morphology and size after sporulating the oocysts [35,36]. To do this, the stool samples were incubated in 2.5% potassium dichromate (K_2_Cr_2_O_7_) at 27 ± 2 °C for 5 days under conditions of constant oxygenation. The sporulated oocysts were concentrated with a saturated NaCl solution. From each sample, 100 sporulated oocysts, taken at random, were measured using a micrometric eyepiece (Leica Microsystems, ASPELAB, Guadalajara, México).

Blood samples were collected by venipuncture of the jugular vein using a vacuum tube needle (Vacutainer^®^, Becton Dickinson, NJ, USA) in tubes containing EDTA anticoagulant and centrifuged at 3000 rpm for 10 min (VE-4000 VELAB^TM^). Plasma was harvested and frozen in Eppendorf tubes at −20 °C for further laboratory processing. The levels of alanine aminotransferase (ALT), aspartate aminotransferase (AST), gamma glutamyl transferase (GGT), alkaline phosphatase (ALP), blood urea nitrogen (BUN), creatinine and urea were measured using a Multiskan FC Photometer microplate reader (Thermo Scientific, Waltham, MA, USA) and BioSystems^®^ reagents (Biosystems S.A., Barcelona, Spain).

### 2.6. Data Analysis

A descriptive analysis of the percentage of presentation of *Eimeria* was performed. Variables consisting of the reduction of coccidial oocysts, productive performance and blood parameter values were compared using an ANOVA to detect statistical differences between groups. Where necessary, the mean contrast was analysed with the Tukey test to specify the statistically different groups. Significant differences were considered when the value of *p* ≤ 0.05. All this was undertaken using the SPSS statistical package, version 20.0 (Statistical Package for the Social Sciences). The analysis of the elimination trend for *Eimeria* oocysts was carried out using polynomial regression.

The following mathematical model was adopted [37]:

Yij = μ + τj + ϵij,

in which Yij is the j-th observation of diet i; μ is the general mean response; τj is the non-random effect of diets, in which Σ_(ⅈ = 1)^k τj = 0; and ϵij = is the random diet error.

## 3. Results

### 3.1. Stool Appearance

The animals that received the seeds presented type II stools, according to the classification by Burke et al. [20], presenting up to a maximum of three defecations in the 5 h after treatment. After this time, the faeces showed a normal consistency.

### 3.2. Parasite Infection Performance

**Figure 1:**
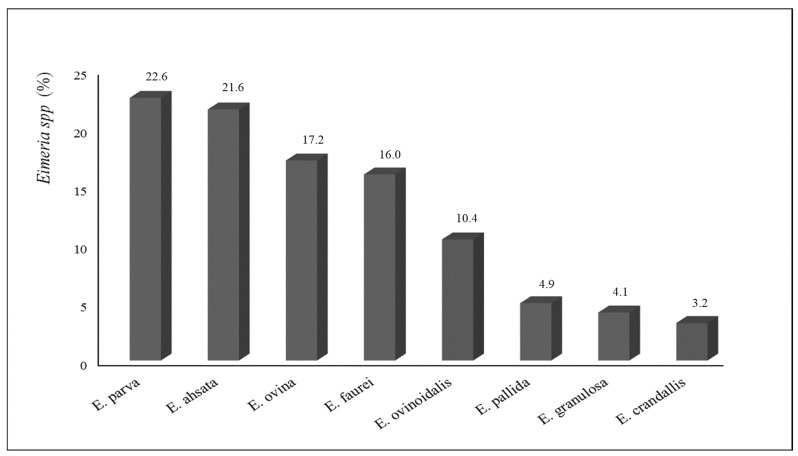
*Eimeria* species identified.

The samples analysed allowed the identification of six species of Eimeria (*E. parva, E. ahsata, E. ovina, E. faurei, E. pallida* and *E. granulosa*), as well as two species considered especially pathogenic (*E. ovinoidalis* and *E. crandallis*) (Figure 1). To differentiate *E. crandallis*, *E. weybridgensis* and *E. ovina*, we took into account the keys indicated by Wright and Coop [36].

**Figure 2:**
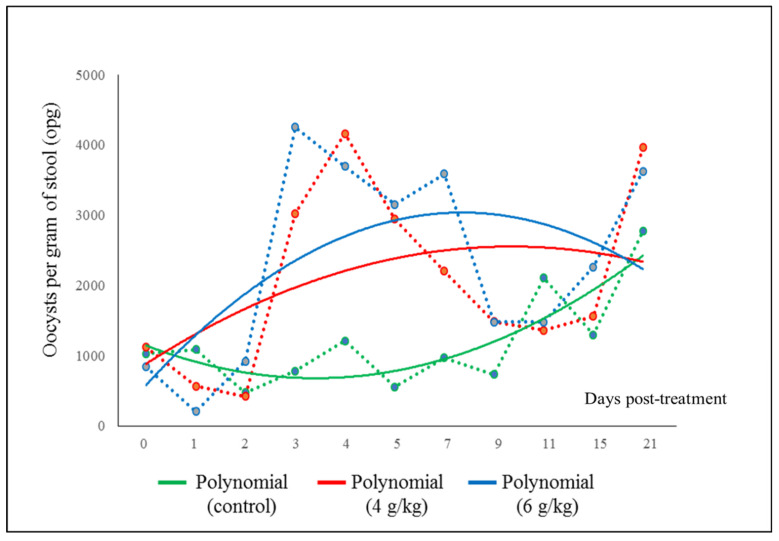
Eimeria oocyst excretion reduction trends for the treatments administered. The dashed line indicates the oocysts eliminated each day (opg) in accordance with the seed dose.

The control group presented a lower elimination of oocysts (*p* < 0.05) compared to those groups that received *Hura crepitans* seeds (Table 2). The greatest elimination of oocysts occurred in group Gr. 2, which received the highest dose of seeds (6 g/kg B.W.), although a significant statistical difference between the two treated groups was not found (*p* > 0.05) (Table 2). The highest oocyst removal occurred between days 5 and 9 in the treated groups; however, the curve marking the trend in oocyst removal was higher in group 2 than in group 1 (Figure 2). 

### 3.3. Productive Performance

Daily feed intake (DFI), total weight gain (TWG), daily weight gain (DWG) and feed conversion efficiency (FCE) were similar between the three groups (*p* > 0.05), as shown in Table 3.

### 3.4. Blood Parameters

The enzymes (ALT, AST, GGT and ALP) and the creatinine analysed were found to be within the normal levels. Only the urea and BUN values were slightly increased around day 7 post-treatment, although without statistical difference (Table 4).

## 4. Discussion

### 4.1. Clinical Signs

The pasty aspect of the faeces was attributed to the latex that is distributed throughout the plant (bark, leaves and seeds) since, in humans, ingesting this seed produces diarrhoea [38]. It should be noted again that the soft consistency of the stool did not last for a long time, which coincides with the observations of Mahmoud et al. [38], who stated that there was a normal return of appetite and stool consistency, although they do not mention the time that elapsed.

The latex of *Hura crepitans* (sandboxtree or possumwood, also called ceiba blanca, ceiba lechosa, tronador, achohó, javillo, tronador, habillo, salvadera, molinillo, arceira, catauá, arbre du diable and posentri, among other names) is considered toxic and highly irritating to the skin and mucosa. In humans, intake of *H. crepitans* seeds produces a burning sensation in the mouth, vomiting, diarrhoea, dyspnoea and headache [39].

However, the lambs that received the different doses of *H. crepitans* did not show any adverse clinical signs. In contrast, muscle tremors, mainly in the head and neck, and uncoordinated gait were described in sheep poisoned by *Ipomoea asarifolia* [40], while tremors, falls, stiff gait, ataxia, muscle spasms and sudden death were cited for goats poisoned by *Marsdenia megalantha* [41].

### 4.2. Parasite Infection Performance

In a study carried out on male and female lambs, nine Eimeria species known to be pathogenic for sheep were identified (*E. ovina*, *E. parva*, *E. faurei*, *E. ahsata*, *E. crandallis*, *E. ovinoidalis*, *E. granulosa*, *E. intrincata* and *E. punctata*), of which the first six coincided with those found by us. This coincidence could be possibly due to the pre-patency periods of species and the differing abilities of oocysts to survive in the environment, pastures and pens [42]. 

In this research, we did not characterize the phytochemical compounds of the *Hura crepitans* seeds. Some previous studies have observed the presence of multiple substances, depending on the part of the tree sampled. Toxalbumins, hurina and crepitin are distributed among all the plant organs [23], while the stem bark and leaves of *H. crepitans* contain tannins, saponins, flavonoids, coumarins, glycosides and triterpenoids, although dafnane diterpenes, daphnetoxin acid and huratoxin were also isolated, along with apocynin and methylpentadecanoate [22]. Phytochemicals, such as glycosides, alkaloids, flavonoids and tannins [21,22]; glycosides and steroids [43]; and crepitin, glucosamine and lectin [21,23], have also been found. 

The greater elimination of oocysts in the days after the administration of seeds can be attributed to the chemical components of the seeds, among which is latex, which Spanó et al. [44] describe as an emulsion mainly used as a means of defence against insects and to control the growth of microbial phytopathogens. This research correlates with that carried out by Mahmoud et al. [38] on the reduction of coccidia. Using four experimental groups (group 1: control, group 2: infected without treatment, group 3: infected and treated with *Calotropis procera* latex and group 4: infected and treated with sulfadimidine), they verified that group 3, expelled more coccidial oocysts than group 4, as also occurred in our research, possibly due to the purgative effect of latex. However, the latex used was obtained from *C. procera*, a North African shrub with sap containing latex, calcium oxalate and other cardioactive substances.

The important reduction in oocysts may also be attributable to condensed tannins, since the *H. crepitans* tree contains these compounds, albeit in low concentrations [45,46], and the effectiveness of condensed tannins in the control of coccidia has been demonstrated in several studies [20,47].

It is possible that the treatment could have been administered before the sporulation of the oocyst and that sporocysts—and, therefore, sporozoites, which are the infecting form—were formed.

It is important to note that the slatted floor could have contributed significantly to the control of Eimeria by keeping the infecting forms away from the animals [48]. For this reason, it would be advisable to carry out another experiment with animals housed on dirt floor to more precisely evaluate the effectiveness of ingesting *Hura crepitans* seeds.

### 4.3. Productive Performance

The daily weight gain, feed intake and feed conversion were similar to those observed by Macias-Cruz et al. [49], who reported values of 170 g/d, 1.2 kg/d and 7.3 kg/kg, respectively, when they used female Pelibuey, Pelibuey × Katahdin and Pelibuey × Dorper sheep and diets with a content of 16.4% of crude protein (CP) and 2.7 Mcal/kg of metabolizable energy (ME) in the finishing period.

Novelo et al. [50] found no differences in dry matter consumption when they compared two groups of grazing goats with different parasite loads, measured by the number of nematode eggs per gram of faeces. Méndez-Ortiz et al. [51] stated that gastrointestinal nematodes negatively influence dry matter consumption but that this impact can be reduced by adding protein and energy to the diet of animals. This may be the reason why there were no differences in the feed consumption of the lambs in the present research.

The observed weight gains were slightly lower than those reported with Pelibuey sheep fed complete diets [52,53]. Bustamante [54] found lower gains (132 g/day/lamb), while the daily weight gain was higher than the values of 124, 158 and 159 g/d reported by Partida et al. [55] with Pelibuey, Pelibuey × Suffolk and Pelibuey × Dorset sheep, respectively. According to Avendaño et al. [56], Pelibuey crossbred lambs and Dorper, or Katahdin, rams show greater weight gains than pure Pelibuey lambs. However, the dry matter consumption (1.03, 1.06 and 1.03 kg/d) and the feed conversion (8.3, 6.7 and 6.5 kg of feed/kg of B.W.) were similar [55]. The differences found could possibly be attributed to the duration of the experiments; the age of the animals; the addition of additives, such as ionophores; and the protein content in the diet [53], although Rivera et al. [42], in a 60-day study, obtained greater total weight gains in those animals with a lower load of coccidial oocysts.

### 4.4. Blood Parameters

Plasma ALT, ASAT, GGT, FA and creatine values were, in general, within the ranges considered normal [57,58]. Increases in these enzymes are generally attributed to liver damage, although they do not always have this origin. Fluctuations in ALT, AST and GGT values may indicate an intensification of metabolic processes [59], allowing us to rule out significant liver injury due to the fact that the enzymes analysed were not manifestly modified and in the same direction. It must be taken into account that the reference values found in the literature were derived from lambs of different breeds reared in different environmental and nutritional conditions, which reduces the precision of the comparison [58].

The uraemia and BUN values were above the reference interval [57,58,60]. Both feeding and handling can influence serum urea levels in ruminants. A higher protein intake causes an increase in blood urea and, therefore, the BUN rate [61,62]. Approximately 70% of the proteins are converted to ammonia in the rumen to be used by ruminal microorganisms for the synthesis of structural proteins. Therefore, degradable rumen protein levels are directly related to the rumen ammonia concentration. A considerable portion is absorbed and, on reaching the liver, converted to urea. Thus, the BUN concentration reflects changes in rumen ammonia production [58]. The increase observed on day 7 is difficult to explain and appears to be a one-time event, since, in our case, the amount of protein in the diet did not change, and the levels quickly returned to the initial values. It may have been due to overnight fasting, since blood samples were taken before the lambs were fed and showed increased tissue catabolism (from body proteins) and haemoconcentration due to lower water intake.

These values can also increase due to kidney damage [27,63], but this is usually accompanied by a parallel increase in blood creatinine levels [63,64]. According to Braun and Lefebvre [63], although no test is sensitive in the early diagnosis of kidney disease due to the large functional reserve of the kidneys, creatinine measurement is the most widely used test to diagnose and monitor kidney function in mammals, as the levels of creatinine increase in renal disease. Serum creatinine levels in lambs raised under tropical conditions have been reported to be lower [58] with respect to the levels indicated by Kaneko et al. [57]. Creatinine values may be slightly altered due to the age of the animals, since, according to Gregory et al. [65], young animals up to 12 months of age have lower serum values than adults. Creatinine levels are not strongly affected by nutrition, although they do increase due to the breakdown of protein stores to meet energy demand [57]. Since creatinine concentrations are associated with the protein status of ruminants and correlate with muscle deposition, creatinine levels tend to increase as animals age [58]. In our case, even though our subjects were young animals, at no time could we see increases in blood creatinine that could indicate alterations in the lambs.

## 5. Conclusions

The administration of *Hura crepitans* seeds at doses of 4 and 6 g/kg B.W. enhanced the elimination of coccidial oocysts during the first 5 to 7 days after their administration, without affecting the productive behaviour or the health of the growing sheep. However, we think it would be very interesting to carry out future investigations isolating and characterizing the main compounds of *Hura crepitans* seeds that act against parasitic protozoa.

## Figures and Tables

**Table 1:** Ingredients and chemical composition of the diet.

Ingredient	Inclusion Percentage ^1^
Cracked corn	22.88
Ground sorghum	22.88
Soybean meal	17.29
Liquid molasses	4.56
Alfalfa hay	17.35
Tallow	0.99
Minerals	2.48
**Chemical composition**	
Dry matter	91.31
Crude protein	17.89
ADF	7.45
NDF	11.93
NME	1.93
NGE	1.20
ME	2.83

Footnotes: ^1^ Percentage in dry matter; ADF: acid detergent fibre; NDF: neutral detergent fibre; NME: net maintenance energy, expressed in Mcal/kg; NGE: net gain energy, expressed in Mcal/kg; ME: metabolizable energy, expressed in Mcal/kg.

**Table 2:** Excretion of coccidial oocysts per gram of faeces (opg) in each experimental group.

Treatment	Average	Stand. Dev.	Minimum	Maximum
**Gr. 1**	1886 ^a^	2280	0	10,650
**Gr. 2**	2134 ^a^	2760	0	13,650
**Gr. 0, Control**	1110 ^b^	1511	0	9500

Treatments—quantity of seeds administered to each experimental group: Gr. 1 (4 g/kg); Gr. 2 (6 g/kg); Gr. 0 (control = 0 g/kg). Different letters in the same column indicate statistical differences (*p* < 0.05).

**Table 3:** Productive performance (means ± SD, in g) of Pelibuey lambs with different doses of *Hura crepitans* seeds.

Treatment	DFI	TWG	DWG	FCE
**Gr. 1**	1089	3.4	0.16	7571
	(205)	(0.9)	(0.04)	(4004)
**Gr. 2**	1103	3.5	0.17	7168
	(216)	(1.1)	(0.05)	(2299)
**Gr. 0, Control**	1131	3.7	0.17	6876
	(143)	(0.8)	(0.37)	(1554)
***p***	0.088	0.893	0.894	0.898

Treatments—quantity of seeds administered to each experimental group: Gr. 1 (4 g/kg); Gr. 2 (6 g/kg); Gr. 0 (control = 0 g/kg). DFI = daily feed intake (g), TWG = total weight gain (kg), DWG = daily weight gain (g), FCE = feed conversion efficiency; (*p* < 0.05).

**Table 4:** Blood parameters (means ± SD) of Pelibuey lambs treated with different doses of *Hura crepitans* seeds.

	Days of Treatment						
		0	1	3	7	8	9
**ALT** (UI/L)	**Gr. 1**	19.9 ± 3.6	20.2 ± 8.3	14.1 ± 5.0	26.9 ± 3.7	15.5 ± 4.9	10.6 ± 4.3
	**Gr. 2**	24.1 ± 8.1	22.4 ± 8.5	17.3 ± 6.2	24.6 ± 5.5	16.9 ± 3.0	3.2 ± 4.7
	**Gr. 0, control**	23.2 ± 8.3	17.9 ± 5.7	14.4 ± 6.4	19.6 ± 9.5	16.6 ± 5.4	13.2 ± 9.3
**AST** (UI/L)	**Gr. 1**	58.1 ± 16.9	80.7 ± 25.7	49.3 ± 21.6	60.2 ± 37.3	11.3 ± 8.2	24.8 ± 11.5
	**Gr. 2**	52.6 ± 8.0	63.3 ± 30.1	57.1 ± 18.7	64.9 ± 15.5	9.2 ± 4.2	20.6 ± 11.9
	**Gr. 0, control**	50.5 ± 19.5	85.6 ± 42.1	60.7 ± 25.0	57.2 ± 13.3	7.9 ± 4.8	13.6 ± 4.5
**GGT** (UI/L)	**Gr. 1**	26.9 ± 9.0	23.5 ± 3.4	31.3 ± 4.5	25.3 ± 18.0	18.9 ± 2.6	31.0 ± 6.3
	**Gr. 2**	27.2 ± 9.9	21.0 ± 13.8	30.3 ± 12.3	19.9 ± 5.5	17.2 ± 2.6	28.9 ± 4.2
	**Gr. 0, control**	25.6 ± 4.7	27.6 ± 6.3	35.6 ± 5.4	19.9 ± 4.5	15.5 ± 9.6	32.3 ± 4.7
**ALP** (UI/L)	**Gr. 1**	123.6 ± 41	236.2 ± 99	81.7 ± 44	136.7 ± 33	133.2 ± 39	337.3 ± 98
	**Gr. 2**	124.1 ± 25	264.4 ± 137	107.8 ± 37	130.9 ± 39	152.1 ± 79	319.8 ± 98
	**Gr. 3, control**	126.3 ± 31	270.5 ± 118	83.7 ± 32	127.2 ± 21	133.6 ± 62	374.4 ± 107
**Creatinine** (µmol/L)	**Gr. 1**	25.7 ± 19.4	11.4 ± 4.4	15.8 ± 14.1	20.9 ± 2.7	5.8 ± 0.9	6.9 ± 0.9
	**Gr. 2**	14.1 ± 11.3	9.4 ± 0.9	12.4 ± 2.7	12.6 ± 8.0	11.1 ± 11.5	8.0 ± 0.9
	**Gr. 0, control**	12.4 ± 5.3	9.6 ± 2.7	10.5 ± 0.9	8.9 ± 0.9	10.2 ± 9.7	7.2 ± 0.9
**BUN** (mmol/L)	**Gr. 1**	2.80 ± 0.3	2.80 ± 0.9	2.60 ± 0.9	8.82 ± 1.0	4.45 ± 0.7	3.13 ± 0.4
	**Gr. 2**	2.83 ± 0.4	3.20 ± 0.3	2.59 ± 0.8	8.26 ± 1.4	4.58 ± 0.5	3.12 ± 0.31
	**Gr. 0, control**	2.67 ± 0.4	3.15 ± 0.7	2.21 ± 0.3	8.70 ± 1.6	4.97 ± 0.9	3.16 ± 0.21
**Urea** (mmol/L)	**Gr. 1**	1.30 ± 0.1	1.30 ± 0.4	1.21 ± 0.4	4.10 ± 0.5	2.07 ± 0.3	1.46 ± 0.2
	**Gr. 2**	1.32 ± 0.2	1.49 ± 0.2	1.21 ± 0.4	3.85 ± 0.6	2.13 ± 0.2	1.45 ± 0.1
	**Gr. 0, control**	1.24 ± 0.2	1.47 ± 0.3	1.03 ± 0.2	4.05 ± 0.8	2.32 ± 0.4	1.47 ± 0.1

Treatments—quantity of seeds administered to each experimental group: Gr. 1 (4 g/kg); Gr. 2 (6 g/kg); Gr. 0 (control = 0 g/kg). ALT = alanine amino transferase, IU/L; AST = aspartate amino transferase, IU/L; GGT = Gamma glutamyl transferase, IU/L; ALP = alkaline phosphatase, IU/L; BUN = blood urea nitrogen, mmol/L. No significant differences were observed (*p* < 0.05).

## Data Availability

Not applicable.
